# Lifestyle-based nomogram for identifying the Chaoshan inhabitants of China at high risk of *Helicobacter pylori* infection

**DOI:** 10.1186/s12876-023-02990-2

**Published:** 2023-10-18

**Authors:** Yi-ting Lin, Pei-ru Wang, Wen-wen Xue, Si-si Zhou, Ze-yu Huang, Yu-ting Li, Zhuo-na Zheng, Wen-jing Hou, Qi-xian Chen, Jing Yu

**Affiliations:** 1 Department of Gastroenterology, The First Affiliated Hospital of Shantou University Medical College, Shantou, 515041 China; 2 Department of Nursing, Shantou Central Hospital, Shantou, China

**Keywords:** Nomogram, Risk factor, *Helicobacter pylori* infection, Chaoshan region

## Abstract

**Background:**

*Helicobacter pylori* (HP) infection is associated with various diseases. Early detection can prevent the onset of illness. We constructed a nomogram to predict groups at high risk of HP infection.

**Methods:**

Patients who underwent regular medical check-ups at hospital in Chaoshan, China from March to September 2022 were randomly allocated to the training and validation cohorts. Risk factors including basic characteristics and lifestyle habits associated with HP infection were analyzed by logistic regression analyses. The independent varieties were calculated and plotted into a nomogram. The nomogram was internally validated by receiver operating characteristic curve, calibration, and decision curve analyses (DCAs).

**Results:**

Of the 945 patients, 680 were included in the training cohort and 265 in the validation cohort. 356 patients in training cohort with positive 13 C-UBT results served as the infected group, and 324 without infection were the control group. The multivariate regression analyses showed that the risk factors for HP infection included alcohol consumption (OR = 1.29, 95%CI = 0.78–2.13, *P* = 0.03), family history of gastric disease (OR = 4.35, 95%CI = 1.47–12.84, *P* = 0.01), living with an HP-positive individual (OR = 18.09, 95%CI = 10.29–31.82, *P* < 0.0001), drinking hot tea (OR = 1.58, 95%CI = 1.05–2.48, *P* = 0.04), and infection status of co-drinkers unknown (OR = 2.29, 95%CI = 1.04–5.06, *P* = 0.04). However, drinking tea > 3 times per day (OR = 0.56, 95%CI = 0.33–0.95, *P* = 0.03), using serving chopsticks (OR = 0.30, 95%CI = 0.12–0.49, *P* < 0.0001) were protective factors for HP infection. The nomogram had an area under the curve (AUC) of 0.85 in the training cohort. The DCA was above the reference line within a large threshold range, indicating that the model was better. The calibration analyses showed the actual occurrence rate was basically consistent with the predicted occurrence rate. The model was validated in the validation cohort, and had a good AUC (0.80), DCA and calibration curve results.

**Conclusions:**

This nomogram, which incorporates basic characteristics and lifestyle habits, is an efficient model for predicting those at high risk of HP infection in the Chaoshan region.

## Key findings

A nomogram, which included alcohol consumption, living with an HP-positive individual, using serving chopsticks, drinking hot tea, a family history of gastric cancer, the infection status of co-drinkers unknown, and drinking tea > 3 times per day, was established to to predict groups at high risk of HP infection in Chaoshan.

**What is known and what is new?**

Numerous logistic regression studies have been conducted to investigate the risk factors for *Helicobacter pylori* (HP) infections. The use of serving chopsticks has been proven to be a protective factor. Our study results were presented in a nomogram. The temperature of the tea, the duration of tea consumption, the frequency of tea consumption, sharing cup use, and the infection status of co-drinkers were considered in this study.

**What is the implication, and what should change now?**

We created a simple tool for predicting those at high risk of HP infection in the Chaoshan region. It also provides a basis for early testing and treatment HP infection.

## Background

Due to its high incidence, *Helicobacter pylori* (HP) infection has become a global burden [1]. It was estimated that approximately 44 billion people worldwide were infected with HP in 2015 [1]. The pooled prevalence of HP is nearly 50% in mainland China [2]. HP plays an important role in gastric disorders, including simple gastritis, peptic ulcer, gastric malignancies [3], and gastric mucosa-associated lymphoid tissue lymphoma [4]. The effects of HP infection are not only restricted to digestive diseases. HP infection is also linked to numerous diseases or conditions, such as diabetes [5], cardiovascular diseases [6], and pregnancy complications [7–10]. However, eradication treatments are still challenging due to antibiotic resistance [11] and an increase in the relapse rate [12]. Thus, there is a need for early detection.

From the 7th national population census data [13], China is home to > 1.4 billion people. Compared to the population growth of 11.1%, the costs associated with HP testing increased by 42.6% between 2013 and 2018 [14]. Thus, the increase in HP-related costs appears to be out of proportion to the population growth. Besides, HP infection occurs in only 26.5% of those with gastrointestinal symptoms [15]. It is not cost-effective to screen the general population in China for HP infection [16]. However, screening in the whole population represents an unrealistic financial burden. Thus, there is an urgent need to screen those at high risk of HP infection.

Epidemiologic studies have identified several factors related to HP infection. In developing countries, the risk factors for HP infection include poverty, overcrowding, and unhygienic conditions [17]. In developed countries, the risk factors for HP infection are related to lifestyle and dietary factors rather than socioeconomic factors [18]. In the adolescent population, age, and a middle school and above level of education were found to be correlated with HP infection [19]. In the adult population, age, education, dietary habits, financial situation, and disease history were found to be independent risk factors for HP infection [20]. The applicability of risk factors differs distinctly in different age cohorts and different regions. To date, no analysis of the risk factors of HP infection in the Chaoshan, China population has been conducted.

This study sought to identify the population at high risk of HP infection using a model based on basic characteristics and lifestyle habits. We also used the area under the curve (AUC), a decision curve analysis (DCA), and calibration curves in a training and validation cohort to examine the performance of the model.

## Methods

### Study population

This study was a case-control study. We calculated our sample using PASS 15 software (NCSS, LLC, Kaysville, UT, USA) based on the pre-experiment results. The sample size was estimated to be 386 cases (power = 0.9, 2-sided test = 0.05, dropout rate = 10%). A total of 1,164 patients who underwent health examinations from March 2022 to September 2022 participated in this study. The study subjects were selected from the Medical Examination Center of the First Affiliated Hospital of Shantou University Medical College, Shantou Central Hospital, and Shantou Chaonan Minsheng Hospital. Because patients with digestive system diseases may adopt different lifestyle habits, participants without digestive symptoms were selected from March to September 2021. To be eligible for inclusion in this study, the patients had to meet the following inclusion criteria: (I) be otherwise healthy and free from clinically significant illness or disease; (II) have had no previous HP infection; and (III) have completely finished the 13 C-UBT and questionnaire. Patients were excluded from the study if they met any of the following exclusion criteria: (I) had digestive symptoms; (II) had taken antibiotics or proton pump inhibitors during the last 2 months; and/or (III) had insufficient information available; (IV) minors (age < 18 years old) were excluded. Finally, 680 patients were included in the training chart, and 265 patients were included in the validation chart. For the training chart, 680 patients were divided into Control group (n = 324, HP negative) and Infected group (n = 356, HP positive) according to the 13 C-UBT results.

All the patients who participated in this study signed the informed consent form, and this study was approved by the Institutional Ethics Board of The First Affiliated Hospital of Shantou University Medical College (No. B-2020-180). All the procedures performed in this study involving human participants were conducted in accordance with the Declaration of Helsinki (as revised in 2013).

### Data collection

General information: All the participants completed a questionnaire that was designed to gather data on the patients’ basic personal characteristics (including age, body mass index (BMI), whether smoking, whether alcohol consumption, family history of gastric cancer, the infection status of other inhabitants, and whether use serving chopsticks) and tea-drinking habits (whether drink tea, the years of drinking tea, the number of days per week to drink tea, the number of times per week to drink tea, whether use sharing cup, the temperature of tea, and the infection status of any co-drinkers). The infection status of co-drinkers is whether the people who drink tea together are positive for HP infection. Smoking is defined as the participants smoking > 4 times per week. Alcohol consumption is defined as the male participants drink > 14 standard cups per week, female participants drink > 7 standard cups per week. Tea drinking is defined as the participants drinking > 1 cup every day for > 1 year. Living with an HP-positive individual is defined as living with a patient who is positive for HP infection.

HP infection status: All the participants completed the 13 C urea breath test (13 C-UBT) (HG-IRIS13C Infrared Spectrometer, Beijing Rich-Force Science & Technology Co. Ltd., Beijing, China).

### Grouping

The patients were randomly assigned to the training or validation cohort using R software (v.4.2.1; R Foundation for Statistical Computing, Vienna, Austria) at a 3:1 ratio. Two experienced gastroenterologists without knowledge of the questionnaire results assessed the 13 C-UBT results to divide the training cohort into the control and infected groups.

### Statistical analysis

Using the Kolmogorov-Smirnov test and normal probability plots, the normality of the continuous variables in the training cohort were assessed. Normally distributed continuous variables were expressed as the mean ± standard deviation (SD). The Student’s *t*-test was used to analyze the normally distributed continuous variables. Categorical variables were expressed as n (%). The chi-square test was used to analyze the categorical variables. A preliminary analysis of relevant factors for H. pylori infection was performed using univariate analysis. Gender, age, BMI, smoking (yes/no), alcohol consumption (yes/no), a family history of gastric disease, multi-person households, the infection status of other inhabitants, whether use serving chopsticks, whether drink tea, the years of drink tea, number of days per week to drink tea, and number of times per day that tea was drunk, whether use sharing cup, whether drink hot tea, and the infection status of the co-drinkers were included in the model. Relevant factors with *P* values < 0.05 identified in the univariate analysis were included in the multivariate logistic regression analysis. The odds ratios (ORs) with 95% confidence intervals (CIs) were estimated. In the multivariate logistic regression analysis, parameters with P values < 0.05 were used to build a nomogram. Model performance was assessed by a receiver operating characteristic (ROC) curve analysis. Calibration curves were generated, and a DCA of the nomogram was conducted to compare the observed probabilities with the nomogram-predicted probabilities. The ROC curves of the validation cohort were used to validate the model. The calibration curve and DCA results for the nomogram were plotted. The statistical analysis was carried out using R version 4.2.1 software (The R Foundation for Statistical Computing, Vienna, Austria; www.r-project.org).

## Results

### Basic characteristics and lifestyle habits in training cohort and validation cohort

A total of 1,164 patients who underwent health examinations from March to September 2022 participated in this study. However, 128 questionnaires were excluded due to incomplete responses, and 72 patients were excluded because they had gastric symptoms, and 19 minors were excluded. The inclusion rate was 81%. A total of 945 patients were included in the final data analysis. The patients who met the inclusion criteria were allocated to the training or validation cohorts at a 3:1 ratio using R software (v.4.2.1; R Foundation for Statistical Computing, Vienna, Austria). A total of 680 patients were included in the training cohort and 265 patients were included in the validation cohort. The result of Table 1 showed that there was no statistically significant difference between the training cohort and the validation cohort in gender, age, BMI, smoking, alcohol consumption, a family history of gastric disease, multi-person households, living with an HP-positive individual, whether use serving chopsticks, whether drink tea, the years of drink tea, number of days per week to drink tea, and number of times per day that tea was drunk, whether use sharing cup, whether drink hot tea, and the infection status of the co-drinkers (P > 0.05).

**Table 1:** Characteristics of training and validation cohorts

Characteristics	Training cohort (n = 680)	Validation cohort (n = 265)	P value
Gender (n, %)			0.35
Male	371 (54.6% )	135 (50.9%)	
Female	309 (45.4%)	130 (49.1%)	
Age (years)	40.35 ± 12.57	40.72 ± 12.43	0.62
BMI (kg/m^2^)	22.51 ± 2.56	22.28 ± 2.43	0.34
Smoker (n, %)			0.49
No	411 (60.4% )	153 (57.7%)	
Yes	269 (39.6%)	112 (42.3%)	
Alcohol consumption			0.48
No	517 (76.0%)	295 (73.6%)	
Yes	163 (24.0%)	70 (26.4%)	
Family history of gastric cancer (n, %)			0.99
No	633 (93.1%)	247 (93.2%)	
Yes	47 (6.9%)	18 (6.8%)	
Multi-person households (n, %)			0.60
No	30 (4.4%)	9 (3.4%)	
Yes	650 (95.6%)	256 (96.6%)	
Living with an HP-positive individual (n, %)			
No	224(32.8%)	87 (32.9%)	
Yes	227 (33.4%)	83 (31.3%)	0.59
Unknown	229 (33.7%)	95 (35.8%)	0.57
Use of serving chopsticks (n, %)			0.83
No	520 (76.5%)	205 (77.4%)	
Yes	160 (23.5%)	60 (22.6%)	
Tea drinking (n, %)			0.95
No	128 (18.8%)	51 (19.2%)	
Yes	552 (81.2%)	214 (80.8%)	
Number of years (n, %)			
0 year	126 (18.5%)	50 (18.9%)	
0–5 years	80 (11.8%)	29 (10.9%)	0.80
≥ 5 years	474 (69.7%)	186 (70.2%)	0.94
Number of days per week to drink tea (n, %)			
0 day	129 (18.9%)	50(18.9%)	
0–3 days	34(5.0%)	14 (5.3%)	0.98
4–6 days	27 (4.0%)	8 (3.0%)	0.61
7 days	490 (72.1%)	193 (72.8%)	0.87
Number of times per day to drink tea (n, %)			0.89
0 time	134 (18.6%)	50 (18.8%)	
0–3 times	298 (43.8%)	117 (44.2%)	0.98
> 3 times	256 (37.6%)	98 (37.0%)	0.90
Using sharing cup (n, %)			0.40
No	250 (36.8%)	89 (33.6%)	
Yes	430 (63.22%)	176 (66.4%)	
Drinking hot tea (n, %)			0.99
No	420 (61.8%)	163 (61.5%)	
Yes	260 (38.2%)	102 (38.5%)	
Infection status of co-drinkers (n, %)			0.95
No	161 (23.7%)	66 (24.9%)	
Yes	13 (1.9%)	3 (1.1%)	
Unknown	506 (74.4%)	196 (74.0%)	

Continuous variables were presented as the mean ± standard deviation. Categorical variables were expressed as n. BMI, body mass index

### The univariate analysis of basic characteristics and lifestyle habits in training cohort

Of the patients in the training cohort, 356 with positive 13 C-UBT results served as the infected group, and 324 without the HP infection served as the control group. The basic characteristics and tea drinking habits of the training cohort are listed in Table 2. We analyzed the associations between the participants’ baseline characteristics and lifestyle habits in a univariate analysis. Age, smoking, alcohol consumption, family history of gastric cancer, living with an HP-positive individual, the use of serving chopsticks, drinking tea, drinking tea > 5 years, drinking tea every day, drinking tea > 3 times per day, the use of sharing cup, drinking hot tea, and not knowing the infection status of co-drinkers were significantly associated with HP infection (Table 2).

**Table 2:** Patient characteristics and P values of univariate analysis

Variables	Control group(n = 324)	Infected group(n = 356)	P value
Gender			0.18
Male	168 (51.85%)	203 (57.02%)	
Female	156 (48.15%)	153 (42.98%)	
Age (years)	38.05 ± 11.86	42.44 ± 12.84	< 0.0001
BMI (kg/m^2^)	22.27 ± 2.57	22.73 ± 2.52	0.02
Smoking			< 0.0001
No	229 (70.68%)	182 (51.12%)	
Yes	95 (29.32%)	174 (48.88%)	
Alcohol consumption			< 0.0001
No	281 (86.73%)	236 (66.29%)	
Yes	43 (13.27)	120 (33.71%)	
Family history of gastric cancer			< 0.0001
No	319 (98.46%)	314 (88.20%)	
Yes	5 (1.54%)	42 (11.8%)	
Multi-person households			0.79
No	15 (4.63%)	15 (4.21%)	
Yes	309 (95.37%)	341 (95.79%)	
Living with an HP-positive individual			
No	196 (60.49%)	28 (7.86%)	
Yes	17 (5.25%)	210 (58.99%)	< 0.0001
Unknown	111 (34.26%)	118 (33.15%)	0.76
Using serving chopsticks			< 0.0001
No	197 (60.80%)	323 (90.73%)	
Yes	127 (39.20%)	33 (9.27%)	
Tea drinking			0.00
No	77 (23.77%)	51 (14.33%)	
Yes	247 (76.23%)	305 (85.67%)	
Number of years			
0 year	75 (23.15%)	51 (14.33%)	
0–5 years	43 (13.27%)	37 (10.39%)	0.24
≥ 5 years	206 (63.58%)	268 (75.28%)	0.00
Number of days per week			
0 day	76 (23.45%)	53 (14.88%)	
0–3 days	17 (5.25%)	17 (4.78%)	0.78
4–6 days	16 (4.94%)	11 (3.09%)	0.22
7 days	215 (66.36%)	275 (77.25%)	0.00
Number of times per day			
0 times	75 (23.15%)	51 (14.33%)	
0–3 times	142 (43.83%)	156 (43.82%)	0.99
> 3 times	107 (33.02%)	149 (41.85%)	0.02
Sharing cup			0.00
No	143 (44.13%)	107 (30.06%)	
Yes	181 (55.87%)	249 (69.94%)	
Hot tea			< 0.0001
No	231 (71.30%)	189 (53.09%)	
Yes	93 (28.70%)	167 (46.91%)	
Infection in co-drinkers			
No	98 (30.25%)	63 (17.70%)	
Yes	8 (2.47%)	5 (1.40%)	0.32
Unknown	218 (67.28%)	288 (80.90%)	< 0.0001

Continuous variables were presented as the mean ± standard deviation. Categorical variables were expressed as n. BMI, body mass index

### Performing multivariate regression analysis to analyze factors of affecting HP infection

We performed a multivariate logistic regression analysis with the factors that were found to be significant (*P* < 0.05) in the univariate analysis. We found that alcohol consumption (OR: 1.29, 95%CI: 0.78–2.13, *P* = 0.03), family history of gastric cancer (OR: 4.35, 95%CI: 1.47–12.46, *P* = 0.01), living with an HP-positive individual (OR: 18.09, 95%CI: 110.29–31.82, *P* < 0.0001), infection status of co-drinkers unknown (OR: 2.29, 95%CI: 1.04–5.06, *P* = 0.04) and drinking hot tea (OR:1.59, 95%CI: 1.01–2.49, P = 0.04) were risk factors for HP infection; whereas using serving chopsticks (OR: 0.30, 95%CI: 0.12–0.49, *P* < 0.0001) and drinking tea > 3 times per day (OR: 0.56, 95%CI: 0.33–0.95, *P* = 0.03) were protective factors for HP infection (Table 3).

**Table 3:** Result of multivariate analysis

Variables	OR	95% CI	P value
Age	1.02	0.99–1.04	0.12
Smoking	1.06	0.98–1.15	0.31
Alcohol consumption	1.29	0.78–2.13	0.03
Family history of gastric cancer	4.35	1.47–12.84	0.01
Living with an HP-positive individual	18.09	10.29–31.82	< 0.0001
Using serving chopsticks	0.30	0.12–0.49	< 0.0001
Tea drinking	0.72	0.26–1.98	0.52
Drinking tea > 5 years	0.78	0.39–1.56	0.48
Drinking tea every day	0.84	0.42–1.68	0.62
Drinking tea > 3 times per day	0.56	0.33–0.95	0.03
Use sharing cup	0.69	0.39–1.19	0.18
Drinking hot tea	1.58	1.05–2.48	0.04
Infection status of co-drinkers unknown	2.29	1.04–5.06	0.04

OR, odd ratio; CI, confidence interval

### Establishing a nomogram to predict HP infection and validating

**Fig. 1:**
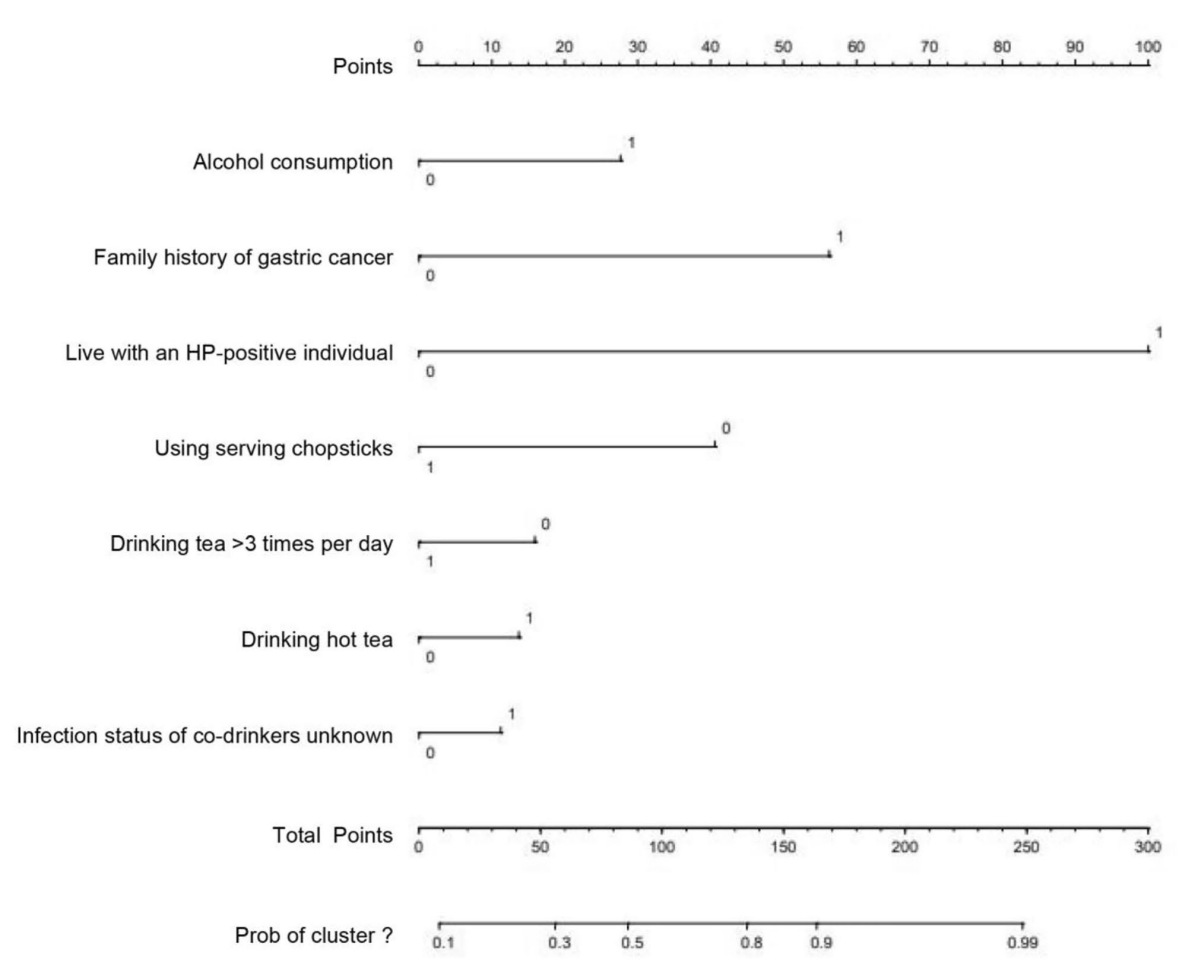
Nomogram for predicting HP infection. The alcohol consumption axis, 0: no; 1: yes; The family history of gastric cancer axis, 0: no; 1: yes; The live with an HP-positive individual axis, 0: no; 1: yes; The using serving chopsticks axis, 0: no; 1: yes; The drinking tea > 3 times per day axis, 0: no; 1: yes; The hot tea axis, 0: no; 1: yes; The infection status of co-drinkers unknown axis, 0: no; 1: yes; HP, *Helicobacter pylori*

**Fig. 2:**
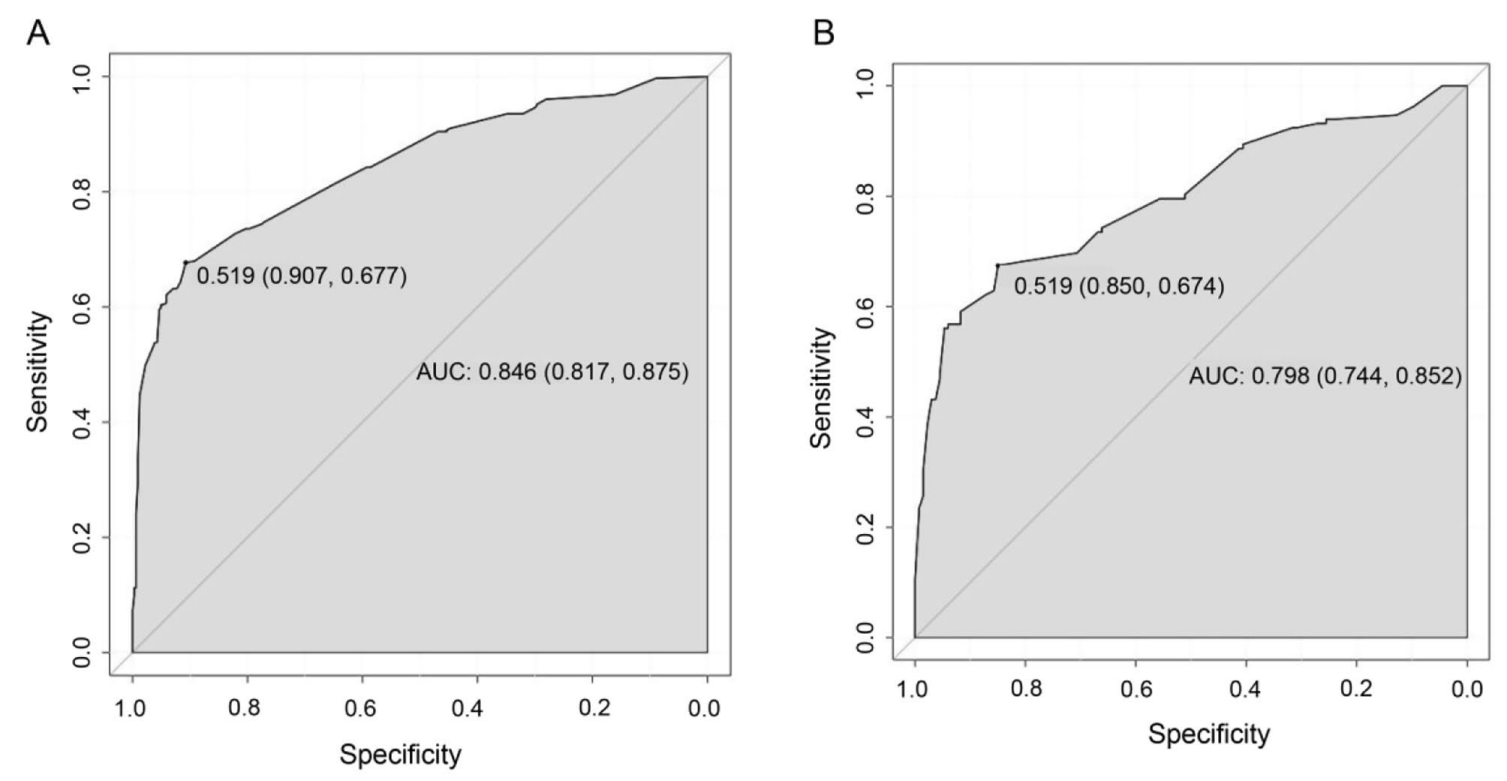
The ROC curve of the nomogram. (**A**) The ROC curve of the nomogram in the training cohort is 0.846 (95% CI: 0.817–0.875), The optimal threshold (0.519) occurs when the sensitivity is 0.677 and the specificity is 0.907; (**B**) the ROC curve of the nomogram in the validation cohort is 0.798 (95% CI: 0.744–0.852). The optimal threshold occurs (0.519) when the sensitivity is 0.674 and the specificity is 0.850. AUC, area under the curve; ROC, receiver operating characteristic

**Fig. 3:**
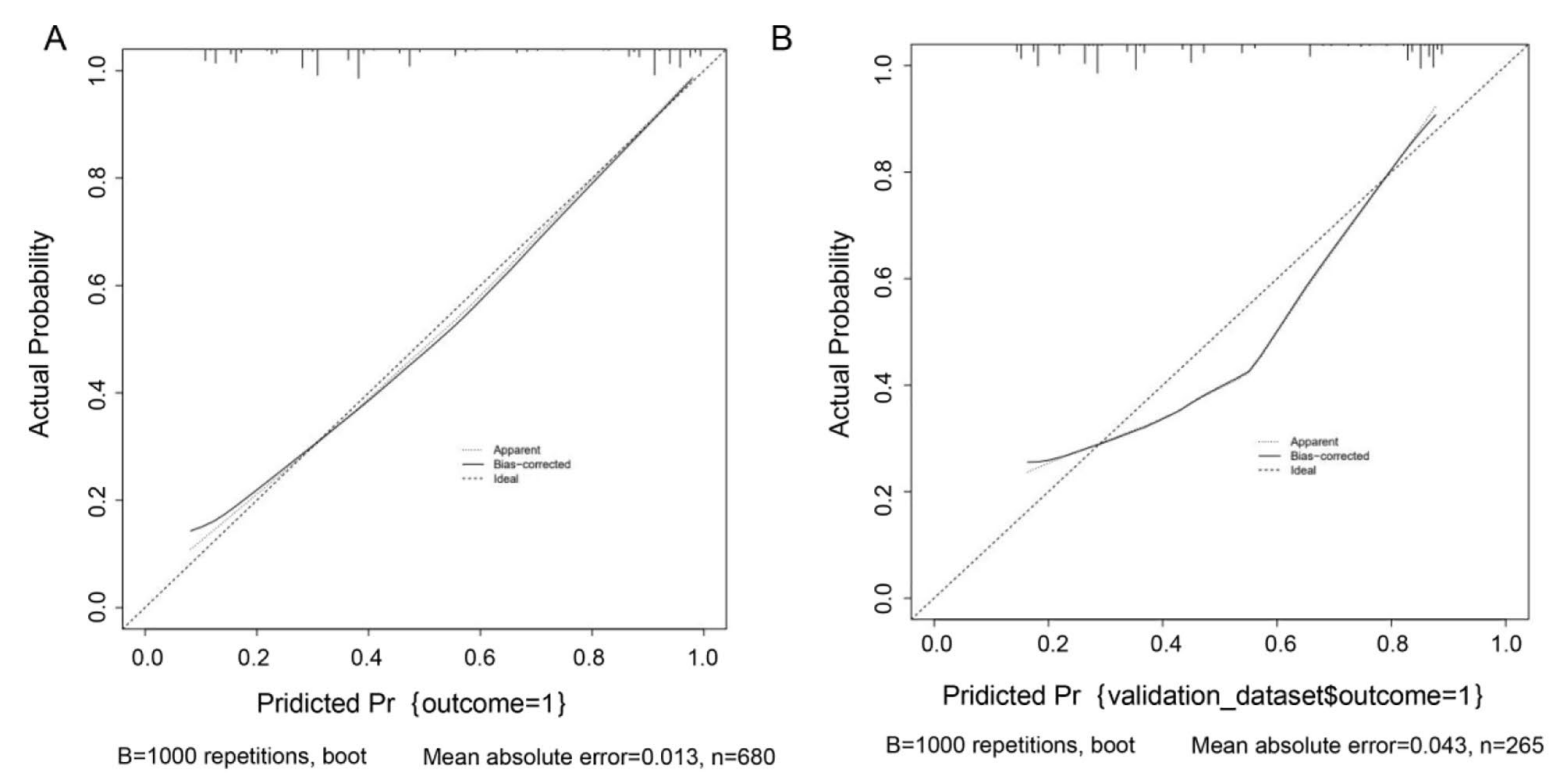
Calibration plots. (**A**) The calibration curve of the nomogram for HP infection risk in the training cohort; (**B**) the calibration curve of the nomogram for HP infection risk in the validation cohort. HP, *Helicobacter pylori*

**Fig. 4:**
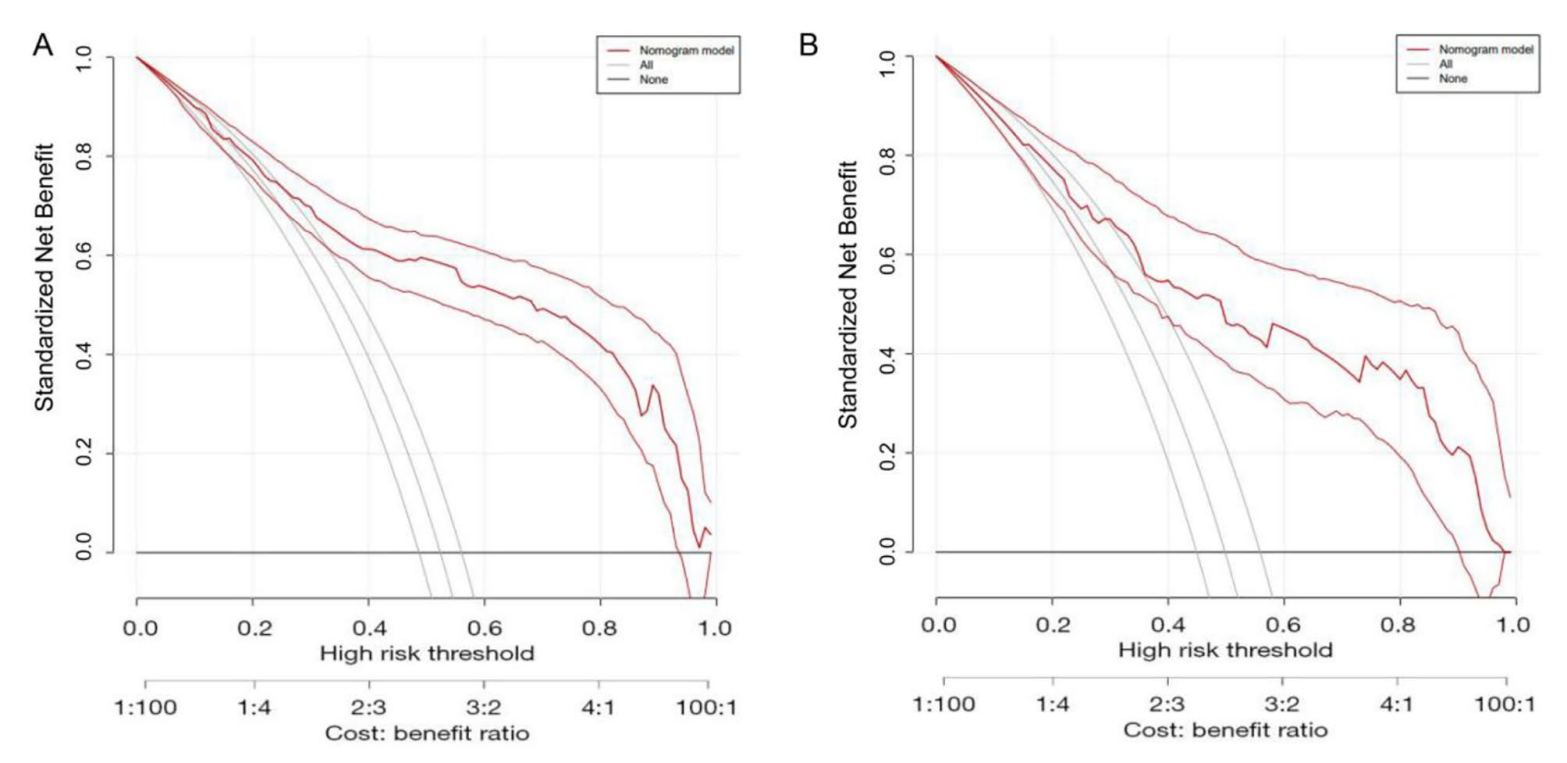
Decision curves of the nomogram. (**A**) Decision curves of the nomogram for HP infection risk in the training cohort; (**B**) decision curves of the nomogram for HP infection risk in the validation cohort. HP, *Helicobacter pylori*

We then established a nomogram for HP infection that included the factors that had been found to have P values < 0.05 in the multivariate analysis (see Fig. 1, in which each factor corresponds to the respective score as numbered on the x-axis). The final risk score was estimated by summing up the individual scores of each factor. A ROC curve of the nomogram was drawn to illustrate its diagnostic ability. As Fig. 2 showed, the nomogram had an AUC of 0.85 (95% CI: 0.82–0.88) in the training cohort and 0.80 (95% CI: 0.74–0.85) in the validation cohort (Fig. 2). For the training and validation cohorts, the calibration curves between the predicted and actual observations were plotted (Fig. 3). The nomogram showed good statistical performance at predicting HP infection. A DCA was conducted to evaluate the efficiency of the nomogram in the training and the validation cohorts (Fig. 4). The nomogram provided clinical net benefits across most thresholds.

## Discussion

Our study found that a number of critical factors predicted HP infection, including alcohol consumption, family history of gastric cancer, living with an HP-positive individual, the use of serving chopsticks, drinking tea > 3 times per day, drinking hot tea, and infection status of co-drinkers unknown. In this study, we established a nomogram for HP infection that included the most relevant factors.

HP infection is associated with many diseases. However, the general awareness of HP infection in general population remains insufficient. In China, the reluctance to be screened is primarily related to being asymptomatic and a lack of knowledge about testing [21]. In a study of the general population, only 16%, 35%, and 43.6% of the subjects correctly answered all the questions asked about HP infectivity, HP harmfulness, and HP preventive measures, respectively [22]. Even the well-educated individuals were reported to have a low overall knowledge level about HP infection [23], and < 10% of the students had a good knowledge level about HP [23].

Screening for HP infection is currently recommended by public health policies. In Japan, due to its cost effectiveness, it is recommended that employees undergo HP screening followed by eradication therapy to prevent gastric cancer [24]. Using the test-and-treat strategy in combination with 13 C-UBT for dyspepsia management and ulcer and gastric cancer prevention is the most cost-effective medical approach [25]. The HP screening and eradication project for school students in Japan has started successfully and shown a steady decrease in the infection rate without major safety concerns [26]. In China, decision trees and Markov models have been developed to evaluate the cost effectiveness of HP screening followed by eradication treatment in asymptomatic Chinese patients, and the results show that for the prevention of gastric cancer, peptic ulcer disease, and non-ulcer diseases, the population-based screen-and-treat strategy was cheaper and more effective than the no-screening strategy [27]. However, a whole population, 13-year follow-up study showed that the HP infection screening and eradication strategy was less effective in terms of quality of life and costs than not screening [28]. Comparisons of participant data and census data, may miss some populations at risk of HP infection risk [29]. Additionally, performing standard tests for serum immunoglobulin G antibodies or 13 C-UBT on the entire population would consume a great deal of resources and have low efficiency. Unnecessary individual screenings are also a waste. Thus, a screening program that identifies those at high risk of HP infection is recommended.

A number of prediction models have been developed to identify individuals at high risk. Using a traditional logistic regression analysis, age, the HP antibody, pepsinogen (PG) I, and PGII were included in a prediction formula [30], which had an AUC of 0.944. Some researchers have developed machine-learning tools to predict HP infection. Logistic regression analysis with K-nearest neighbor (KNN), Least Absolute Shrinkage and Selection Operator (LASSO), support vector machine (SVM), random forest (RF), naive Bayes (NB), and XGBoost (XGB) algorithms have been used to predict HP infections based on lifestyle, behavior, socioeconomic, hygiene, and sanitation factors [31]. AUCs of 0.76–0.79 have been achieved by XBG, NB, RF, SVM, KNN, and LASSO algorithms [31]. Despite being valid, the screening tools developed in these studies employed some parameters that are more difficult to obtain in the general population. Additionally, these models were not intuitive, and conclusions cannot be drawn immediately. Thus, we developed a nomogram that is simple, intuitive, visual, and easy to use.

Tea is one of the most popular and broadly consumed beverages in the world. Tea constituent ions exhibit strong antioxidant, anti-inflammatory, and anti-tumor properties [32]. Drinking tea (OR = 0.26) was found to exert a protective effect against gastric cancer [33]. It was demonstrated that tea polyphenols (the bioactive ingredient of tea) inhibited HP infection and have inhibit antimicrobial [34]. But, a study conducted in Malaysia reported that frequent consumption of tea was a negatively correlated variable (OR = 0.023) for HP infection [35]. These contradictory results may be due to unhealthy tea drinking habits. In the Chaoshan region, drinking tea is a popular lifestyle habit, and resident often share cups during drinking. Thus, our study specifically focused on the tea-drinking habits of the Chaoshan population, such as the number of years, number of days per week, number of times per week tea was drunk, sharing cup, drinking hot tea, and the infection status of co-drinkers. We found that drinking hot tea were risk factors for HP infection, and drinking tea > 3 times per day were protective factors for HP infection. It suggested that the tea-drinking habit effect on HP infection is complicated, and it may be affected by tea consumption and temperature. More research is needed to understand the relationship between tea and HP infection.

The 13 C-UBT was used as a diagnostic tool for the screening of HP infection in this study. When introducing a screening approach, a balance between sensitivity and specificity, cost, invasion, and simplicity should be considered when considering a screening approach [36]. The 13 C-UBT is a method with high sensitivity 97% (95% CI: 96–98%) and specificity 96% (95% CI: 95–97%) [37]. Currently, the 13 C-UBT is the diagnostic tool recommended for the detection of HP infection [38]. Conversely, the sensitivity and specificity of serological tests are relatively deficient. Serological tests have moderately high accuracy (61%), sensitivity (88.37%), and specificity (40.35%) [39]. In addition, positive serologies not only indicate active infection, but also previous infection, and non-specific cross-reacting antibodies. Antibody titers are rarely sufficiently increased to be detected at the early phase of infection [40], which leads to false negative or false positive results. Further, different serology sensitivities are observed across age groups, as the levels of antibodies are lower in children than adults [41].

A logistic regression analysis and nomogram were used to develop this screening tool. Since lifestyle habits can be used to screen high-risk subjects for HP, even community residents can easily determine the risk of HP infection using the nomogram. The nomogram is based on the 5 basic factors listed below. We found the infection status of other inhabitants is a significant independent risk factor for HP infection. This finding is consistent with that of other research. In a retrospective study at a tertiary referral center of Apulia, HP positivity was significantly associated with HP positive relatives [42]. Further, a meta-analysis reported that HP infection was significantly correlated with having a sibling or siblings infected with HP (OR = 3.33, 95% CI: 1.53–7.26) in children aged ≤ 18 years [43]. Multivariate logistic regression analysis of using serving chopsticks as a protective factor against HP infection. This may be because transmission from person to person mainly occurs through fecal-oral or oral-oral routes [44, 45]. The use of serving chopsticks helps to interrupt this transmission route. We found that the risk of HP infection increased with a family history of gastric disease. This finding is contrary to previous studies, which have suggested that a family history of gastric disease does not affect the infection rate [46]. This difference in findings may have arisen, as we excluded patients with gastric symptoms. Patients with gastric symptoms always have a history of gastric disease. This led to different sample sizes of patients with a family history of gastric disease between these two study. We also found that drinking hot tea may be a risk factor for increased HP infection. The consumption of high-temperature foods can disrupt mucosal barriers and lead to the progression of HP infection (OR = 1.32, 95% CI: 1.03–1.69) [18]. Drinking tea > 3 times per day reduces the possibility of HP infection. However, it was only when the consumption of tea was > 3 times a day, which is similar to the Brinkman index [47], that the relationship between drinking tea and HP infection was valid.

This study had a number of limitations. The model was based on individuals from the Chaoshan region. Thus, its applicability to populations of different regions is uncertain, and studies with other populations need to be conducted to determine its applicability. Second, there are still some shortcomings in determining the predictability of the model by randomly selecting an internal validation cohort. The model requires further external verification using large-scale cohort research.

The tool our study developed will help Chaoshan inhabitants to evaluate the risk of HP infection simply by considering their lifestyle habits. High-risk groups can seek timely medical care to test and treat HP infection. Screening high-risk groups for HP infection will save manpower, financial, and medical resources. In addition, HP infection could induce various gastric diseases, including gastritis, gastric ulcers, and gastric cancer. Early prediction and discovery would contribute to controlling the progression of these disease.

## Conclusions

In summary, using lifestyle habits, we created a simple tool for predicting those at high risk of HP infection in the Chaoshan region. Our findings will not only help Chaoshan inhabitants to assess their risk of HP infection easily, quickly and non-invasively, but will also provide a basis for early testing and treatment.

## Data Availability

The datasets generated and/or analyzed in the experiment are available from the corresponding author on reasonable request.
